# Improving the post-acute care discharge score (PACD) by adding patients’ self-care abilities: A prospective cohort study

**DOI:** 10.1371/journal.pone.0214194

**Published:** 2019-03-28

**Authors:** Daniel Koch, Philipp Schuetz, Sebastian Haubitz, Alexander Kutz, Beat Mueller, Helen Weber, Katharina Regez, Antoinette Conca, for the Triage Study Group

**Affiliations:** 1 Department of clinical nursing science, Kantonsspital Aarau AG, Aarau, Switzerland; 2 University Department of Internal Medicine, Kantonsspital Aarau AG, Aarau, Switzerland; University of Virginia, UNITED STATES

## Abstract

**Background:**

Reducing delays in hospital discharge is important to improve transition processes and reduce health care costs. The recently proposed post-acute care discharge score focusing on the self-care abilities before hospital admission allows early identification of patients with a need for post-acute care. New limitations in self-care abilities identified during hospitalization may also indicate a risk. Our aim was to investigate whether the addition of the post-acute care discharge score and a validated self-care instrument would improve the prognostic accuracy to predict post-acute discharge needs in unselected medical inpatients.

**Methods:**

We included consecutive adult medical and neurological inpatients. Logistic regression models with area under the receiver operating characteristic curve were calculated to study associations of post-acute discharge score and self-care index with post-acute discharge risk. We calculated joint regression models and reclassification statistics including the net reclassification index and integrated discrimination improvement to investigate whether merging the self-care index and the post-acute discharge score leads to better diagnostic accuracy.

**Results:**

Out of 1342 medical and 402 neurological patients, 150 (11.18%) and 94 (23.38%) have reached the primary endpoint of being discharged to a post-acute care facility. Multivariate analysis showed that the self-care index is an outcome predictor (OR 0.897, 95%CI 0.864–0.930). By combining the self-care index and the post-acute care discharge score discrimination for medical (from area under the curve 0.77 to 0.83) and neurological patients (from area under the curve 0.68 to 0.78) could be significantly improved. Reclassification statistics also showed significant improvements with regard to net reclassification index (14.2%, p<0.05) and integrated discrimination improvement (4.83%, p<0.05).

**Conclusions:**

Incorporating an early assessment of patients’ actual intrahospital self-care ability to the post-acute care discharge score led to an improved prognostic accuracy for identifying adult, medical and neurological patients at risk for discharge to a post-acute care facility.

## Introduction

Discharge delays lead to unnecessary additional hospital days and are therefore an important cost-factor for acute-care institutions with payment based on diagnosis-related groups [1–4]. Delayed discharge may have grave consequences for patients such as increased risk for infection, pressure ulcer and adverse drug reaction [5]. As recently reported from a Swiss tertiary care hospital, a substantial part (16.4%) of the length of hospital stay is caused by delay in discharge process [4]. In order to keep the waiting time until discharge as short as possible, it is essential to appropriately identify patients with the risk of leaving to a post-acute care (PAC) facility as early as possible. This allows early discharge planning and thus reduction of delayed hospital discharge. For this purpose, the screening instrument post-acute care discharge score (PACD) was recently proposed [6]. In order to further improve the diagnostic accuracy of this instrument, we searched for appropriate predictors.

Little is known about predictors for a PAC facility discharge. Only a few studies investigated this subject, reporting predictors such as age, gender, the presence of geriatric problems, cognitive status, functional ability and dependence at the time of admission [7–9]. Based on theoretical considerations, we searched for studies with endpoints similar to discharge to a PAC facility (e.g. non-home discharge, relocation risk, non-routine discharge planning, self-care limitations and readmission). The studies we found showed that limitations in self-care abilities (e.g. low functional status, poor cognition [10], self-related walking limitations [11–13], or impairment in activities of daily living [14]) are associated with discharge destination [9]. Some of these self-care abilities are already assessed in many hospitals within standardized routine nursing assessments (e.g. the Barthel Index). At the Cantonal Hospital Aarau, Switzerland (KSA), the self-care index (SPI) is used to assess patients’ self-care abilities [15]. Currently the SPI is not validated as a tool to identify patients at risk for discharge to a PAC facility. [15]. Based on the found literature we hypothesized that the SPI, in combination with an established screening instrument (PACD), may help to identify patients at risk for a PAC facility discharge. The term "risk" refers to delayed hospital discharge. The transfer to an aftercare institution does not represent a risk for the patient per se.

We have studied the instruments and their components used in previous studies on this topic. These instruments were: the Barthel Index [9], the Oncology Group performance status (ECOG) [10], the Modified Rankin Scale [11], the “6-Clicks” basic mobility and daily activity measures [12] and the “screening sheet at admission: SSA” [14]. All of these instruments measure the same construct as the SPI, namely patients’ self-care abilities and care dependency. We therefore assume that the SPI is an appropriate instrument to further improve the performance of the PACD.

The aim was to better predict post-acute care needs for patients at the earliest point after an admission through the emergency department (ED). In a large and unselected population of adult patients treated on the wards of mixed internal medicine and neurology, we investigated whether the addition of the scores (PACD and SPI) improves the prognostic accuracy of the screening tool PACD at the KSA for identification of adult hospitalized patients at risk for discharge to a PAC facility.

## Materials and methods

### Design

The Present study is part of the Triage study, a prospective, observational cohort study initially designed to understand the value of admission biomarkers to predict later adverse outcomes (trial registration: ClinicalTrials.gov Identifier, NCT01768494. Registered January 9, 2013) [16]. Results of the Triage project, referring to the PACD as well, have already been published [6, 17].

For the present research question, we consecutively included all medical patients (>16 years) presenting from February 2013 to October 2013 to the ED, KSA. “Medical patient” was defined as a patient with an initial predominant medical/neurological health issue, as judged by the triage nurse [18]. Patients who were transferred from or to another hospital, transferred from a nursing home, died during hospitalization, or had incompletely assessed PACD/SPI data were excluded from the study.

We defined patient’s discharge destination (PAC facility or home discharge) as our main outcome. PAC facility discharge was defined as discharge to a PAC facility following hospital discharge. This includes institutionalized transition care, convalescent care, nursing homes, inpatient rehabilitation center and residential care homes.

### Setting and data collection

The KSA is a 600-bed tertiary hospital and the major urban teaching hospital in the Canton of Aargau, Switzerland. Every year, approximately 6000 inpatients are treated at the internal medicine ward [19]. Upon patients’ admission to the ED, the PACD was assessed by nurses and physicians. Uncollected PACD were retrospectively assessed in the medical ward by the nursing staff. On the ward, nursing staff assessed the SPI within 24 hours after admission, as part of the clinical routine work. All patients were contacted 30 days after hospital admission for a telephone interview with a predefined questionnaire by study-nurses, to assess post-discharge residence. In the case of cognitively impaired patients, their relatives or their care teams were interviewed. Asylum seekers and prison inmates were excluded because they are difficult to reach by telephone. Data on post-discharge residence were also collected from electronic patient records of the medical coding department. A detailed description of the Triage-project was published [20].

### Ethics approval and consent to participate

The institutional Review Board of the Canton Aargau (“Ethikkommission Nordwest- und Zentralschweiz EKNZ”) approved the study and waived the need for informed consent, due to the study design (observational quality control study); EK 2012/059.

### Instruments

#### PACD (KSA version)

The PACD in the KSA version is assessed by four patient-related questions on medical acuity, age, pre-admission living situation and (instrumental) activities of daily living (Table 1). The sum of all subscores then results in a total score. According to Conca, et al. [6, 17], an increased risk of discharge to a PAC facility was defined by a threshold of 8 or more points [21].

**Table 1:** PACD (KSA version).

PACD item	Scoring	Additional information
Number of medically active problems on admission	One point per diagnosis on International Classification of Diseases 10^th^ Revision [22], (Louis Simonet, personal communication on 17.05.2010)	Only diagnoses with diagnostic and/or therapeutic consequence for actual treatment and/or monitoring needs beyond routine count as an active medical problem.
Availability of a person in the same household who can provide help	yes = 0 points; no = 4 points	-
Number of disabilities	1 point per disability = up to 12 points maximum, no disability = 0 points	*Assessed disabilities in (instrumental) activities of daily living*: feeding, grooming, dressing, toileting, bathing or taking a shower, walking, transferring, travelling via car or public transportation, food or clothes shopping (regardless of transport), meal preparation, housework, and medication use (preparing and taking correct dose)
Patient’s age	1 point per decade from 60 years	≥ 100 = +5 ≥ 90 = +4 ≥ 80 = +3 ≥ 70 = +2 ≥ 60 = +1

Abbreviations: PACD: post-acute care discharge score

The PACD score made an accurate prediction within the developers’ validation (area under the curve [AUC]: 0.81) [7]. Similar accuracy was measured in the KSA validation study for medical (AUC: 0.77; sensitivity: 72.6%; specificity: 66.5%) and neurological patients (AUC: 0.68; sensitivity: 41.4%; specificity: 81.4%) [6].

#### SPI

The SPI is a score including 10-items from the 52-items of the “result-oriented nursing-assessment acute care” (ePA-AC version 1.0) [23]. The ePA-AC is used to measure the ability and impairments of a patient, in point values 1–4 (no ability / severely impaired ability / low impaired ability / full ability). The assessment takes place within 24 hours after admission to the ward. The SPI itself is used to predict post-acute care deficit and to serve as an indicator for the severity of nursing dependency [23]. A SPI-score of 10 indicates full dependence, and a score of 40 demonstrate full independence. The cut-point was set on ≤ 32 points by the developers. See Table 2.

**Table 2:** The self-care index (SPI).

**Point value: 1–4** (no ability / severely impaired ability / low impaired ability / full ability)	**Dimension**	**Additional information**
	movement	(e.g. from bed to chair)
	personal hygiene upper body	
	personal hygiene lower body	
	dressing upper body	
	dressing lower body	
	eating food	
	drinking liquids	
	excretion urine	
	excretion stool	
	cognition/consciousness	(ability to acquire knowledge)
**Scoring (sum points):**	From 10 points (full dependency) until 40 points (full independency)	

In a validation study (n = 620), the SPI presented a sensitivity of 85.5% and a specificity of 92.3% for a post-acute care deficit (involvement of nursing case management was defined as interested outcome) and a substantial inter-rater reliability (Cohen’s kappa >0.6) [15]. Another study conducted by the developer confirmed sufficient sensitivity to measure change, construct validity and criterion validity [24]. There is no data about SPI’s accuracy, predicting patients at risk for a PAC facility discharge (Dirk Hunstein, personal communication on 31.03.2017).

### Statistical analysis

Our primary endpoint is discharge to a PAC facility opposed to discharge home. Our main predictors are the PACD and SPI total scores. Discrete variables are expressed as frequency (percentage) and continuous variables as medians and interquartile ranges (IQR). Differences between groups were tested with Pearson’s chi-square test for dichotomous and categorical variables and t-test for the continuous variable age. We report odds ratios $\left(\mathrm{OR}=e^{\hat{\beta }}\right)$ and 95% confidence interval (CI) as measures of association.

To study associations of our predictors and outcome, we calculated logistic regression models with receiver operating characteristic curves (ROC) and the area under the receiver operating characteristic curve (AUC) as a measure of discrimination [25]. To study the incremental benefit of adding the total score SPI to the total score PACD, we calculated joint regression models (the independent variables total PACD score and total SPI score have been centered and then entered in a single step) and report AUC as a mean of discrimination, as well as reclassification statistics [26]. This analysis used continuous variable information with evaluation of the effects on risk category reclassification for patients with or without reaching our primary endpoint. Reclassification to a higher risk group was considered upward movement in classification for patients reaching the endpoint. On the other hand, reclassification downward was considered as a failure for patients reaching the endpoint and vice versa. Improvement in reclassification was estimated by taking the sum of differences in proportions of individuals reclassified upward minus the proportion reclassified downward for patients reaching the endpoint, and the proportion of individuals moving downward minus the proportion moving upward for patients not reaching the endpoint. We calculated the net reclassification improvement (NRI) which assesses improvement in reclassification over risk categories; we also assessed integrated discrimination improvement (IDI), which can be viewed as a continuous version of the NRI without the recourse to a priori defined risk categories. We included 1744 patients (1342 medical and 402 neurological) for the analysis (see Table 3). For internal validation we bootstrapped (1000 replications) all of the performance estimates according to Harrell et al. [27]. This will provide a robust estimate of model performance in the development sample.

**Table 3:** Patient characteristics.

Characteristics	Medical patients			Neurological patients		
	Total	PAC facility discharge	Home discharge	Total	PAC facility discharge	Home discharge
Number of patients	N = 1342	N = 150	N = 1192	N = 402	N = 94	N = 308
Age: median (IQR)	69 (57–78)	77.5* (69–83)	68* (56–77)	69 (55–78)	76* (65–81)	66* (52–77)
Number of men (%)	58.7	39.3*	61.2*	55.5	54.3	55.8
Discharge from						
hospital to (%):						
Home	88.8	0	100	76.6	0	100
Nursing home	4.3	38		1.3	5.3	
Home for the elderly	1.1	10		0.3	1.1	
Rehabilitation	5.8	52		21.9	93.6	
ICD-10 main diagnosis (%):						
Infectious and parasitic						
diseases	13.7	14*	13.7*	3.48	2.1*	4.0*
Diseases of the respiratory						
system	12.3	12.7*	12.3*	0.25	0*	0.3*
Diseases of the circulatory						
system	27.1	21.3*	27.9*	38.5	73.4*	27.9*
Diseases of the digestive						
system	11.3	5.3*	12.1*	.5	1.1*	0.3*
Neoplasms	10.4	8*	10.7*	1.7	0*	2.0*
Diseases of the nervous						
system	0.7	3.3*	0.3*	37.8	16.0*	44.5*
Others	24.5	35.4*	23*	17.8	7.4*	21*
Length of hospital stay (days):						
median (IQR):	6 (4–9)	14* (10–21)	5* (3–8)	6 (3–10)	14* (10–15)	5* (3–7)
PACD (Score) median (IQR)	6 (3–10)	10.5* (7–15)	5* (3–9)	4 (2–7)	5.5* (3–9)	3* (2–6)
SPI (Score) median (IQR)	38 (34–40)	30* (25–35)	38* (35–40)	38 (32–40)	30* (20–37)	39* (35–40)

* Statistically significantly different (PAC facility vs. home discharge); p<0.05

Abbreviations: ICD: International Classification of Diseases 10th Revision; IQR: interquartile range; N: Numbers; PACD: post-acute care discharge score; SPI: self-care index; PAC: post-acute care

Statistical analysis was performed using SPSS 23 of the International Business Machines Corporation (IBM) and STATA 12.2 (StataCorp, College Station, TX, USA). A p-value of <0.05 was considered significant.

## Results

### Characteristics of participants

**Fig 1:**
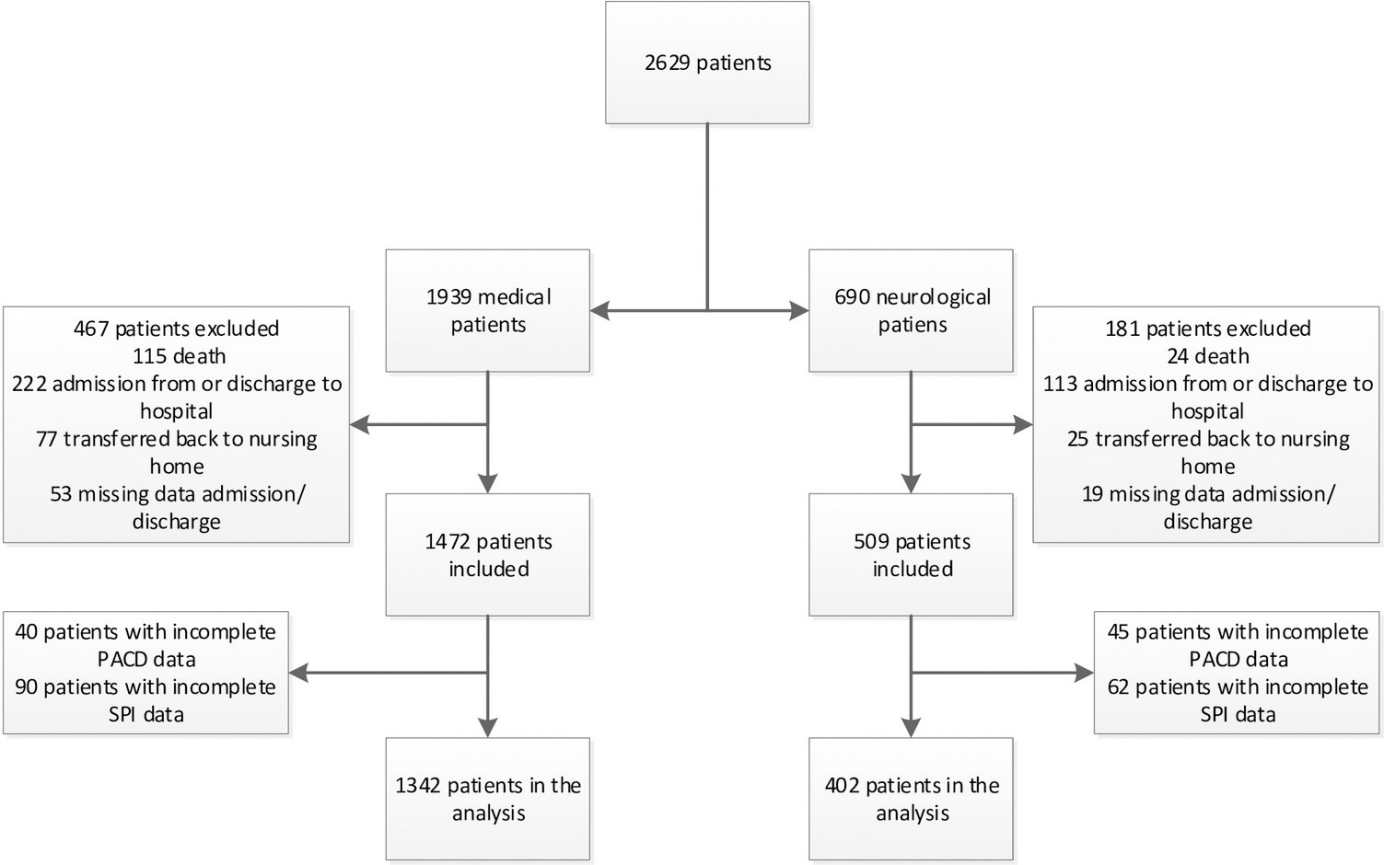
Flow-Chart recruiting process. **There were 1342 medical and 402 neurological patients included in the analysis from a possible 2629 patients.** Abbreviations: PACD: post-acute care discharge score; SPI: self-care index.

During the period of data collection, 2629 medical and neurological patients were screened, and a 30-day interview was performed. We excluded 579 medical and 288 neurological patients due to predefined exclusion criteria. The recruiting process is shown in a flow-chart (Fig 1). The final sample consisted of 1342 medical and 402 neurological patients both with a median age of 69 years (interquartile range [IQR] medical patients 57–78; IQR neurological patients 55–78), a median PACD score of 6 (IQR 3–10) and 4 (IQR 2–7) and both with a median SPI score of 38 (IQR medical patients 34–40; IQR neurological patients 32–40). Transfer destinations for medical and neurological patients to a PAC facility were 11.18% and 23.38% respectively. Due to this large difference and to the fact that neurological patients are more often than medical patients acutely limited in their self-care abilities, we have stratified by medical and neurological diagnosis to ensure applicability of a combined score in both groups.

Patients discharged to a PAC facility compared to home-discharge were in both groups (medical/neurological patients) significantly older, had a longer hospital stay, a higher PACD and a lower SPI. Further sociodemographic data of the patients included is shown in Table 3.

### Combined score (PACD / SPI)

**Fig 2:**
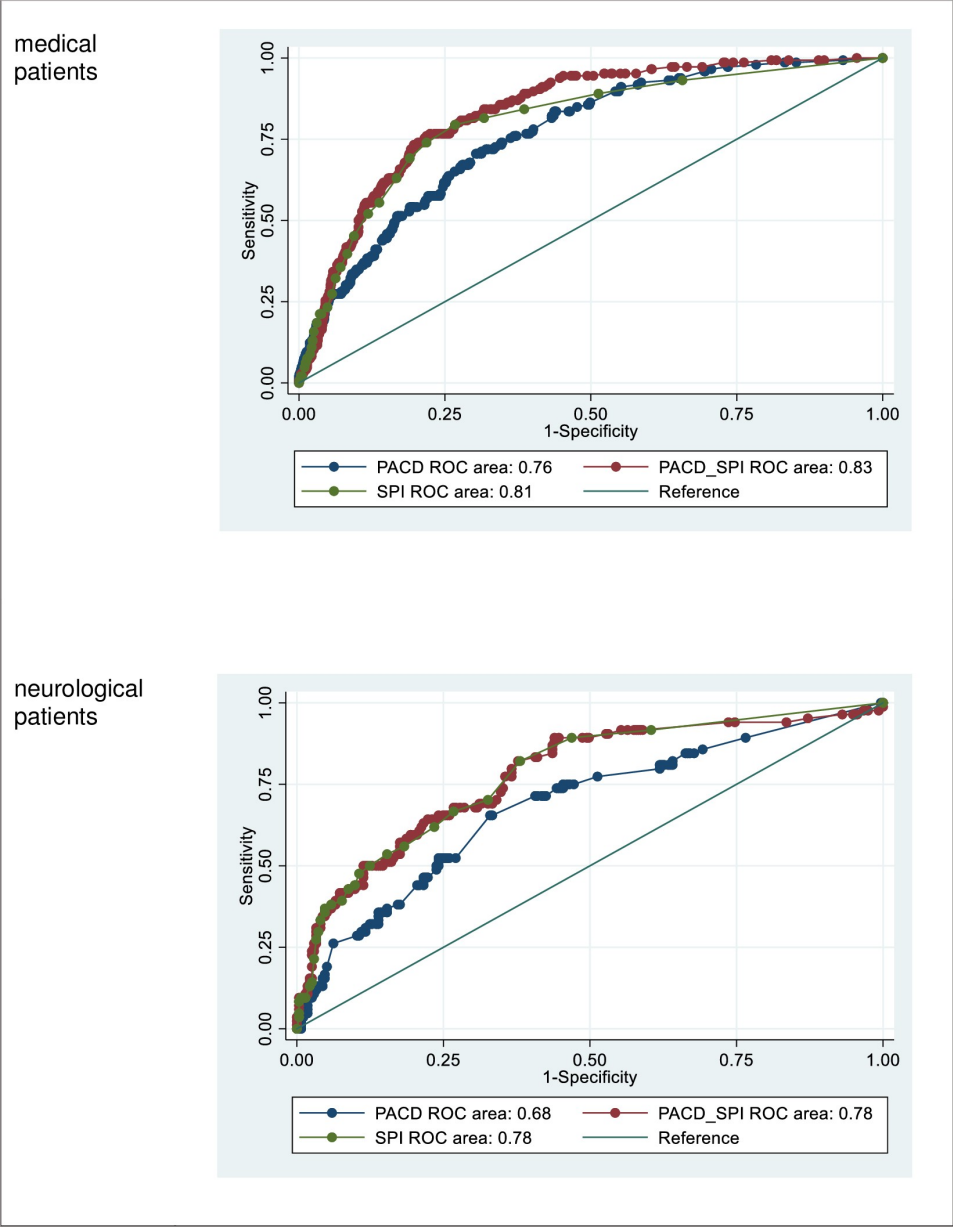
Receiver operating characteristic (ROC) analysis of PACD, SPI and PACD combined with SPI (PACD_SPI) for both fields. Abbreviations: PACD: post-acute care discharge score; ROC: Receiver operating characteristic; SPI: self-care index.

As pictured in Fig 2 significant differences (p<0.01) are shown by comparing the AUC of the medical patients’ PACD (0.760; 95% CI 0.720–0.800) and the PACD combined with SPI (0.833; 95% CI 0.804–0.864) as well as by comparing the neurological patients’ PACD (0.683; 95%CI 0.617–0.750) and PACD combined with SPI (0.778; 95%CI 0.719–0.837).

The model parameters of the logistic regression combining PACD and SPI on the dependent binary outcome discharge to PAC facility is shown within Table 4.

**Table 4:** Model parameters of logistic regression on the combination of PACD and SPI.

Parameter	Odds Ratio (95% CI)	Coefficient (95% CI)	Std. Error	p-value
PACD	1.087 (1.038–1.137)	0.083 (0.036–0.130)	0.022	<0.001
SPI	0.897 (0.864–0.930)	-0.109 (-0.141 - -0.075)	0.013	<0.001
Constant	0.087 (0.070–0.108)	-2.439 (-2.655 - -2.223)	0.010	<0.001

Abbreviations: 95% CI: 95% confidence interval; PACD: post-acute care discharge score; SPI: self-care index

According to the coefficients (Table 4) obtained, the scoring system was established as follows: PACD (coefficient = 0.083): 0.8xPACD; SPI (coefficient = -0.109): 1.1xSPI which results in the combined score: 44+(0.8xPACD)—(1.1xSPI) = combined score. To get a score in the positive range in a similar range like the original score we added 44 and multiplied the coefficients by ten.

By using the probabilites for the main outcome, based on clinical considerations, we generated two thresholds for the combined score and thereby three risk groups. With the upper threshold value, high-risk patients should be identified. The identified patients would automatically be subjected to a more complex screening procedure by the social services. Due to scarce resources, we decided to set the threshold value so that the risk of leaving to an aftercare institution is high (mean probability of around 50%) and thus the more time-consuming clarification by the social service is indicated as often as possible (low number of false positives). With the lower threshold, we have identified patients with very little or no risk (mean probability of around 5%). All patients between the upper and lower threshold have a medium risk (mean probability of around 20%). These patients are examined more closely by the treatment team without the social services being automatically involved.

**Fig 3:**
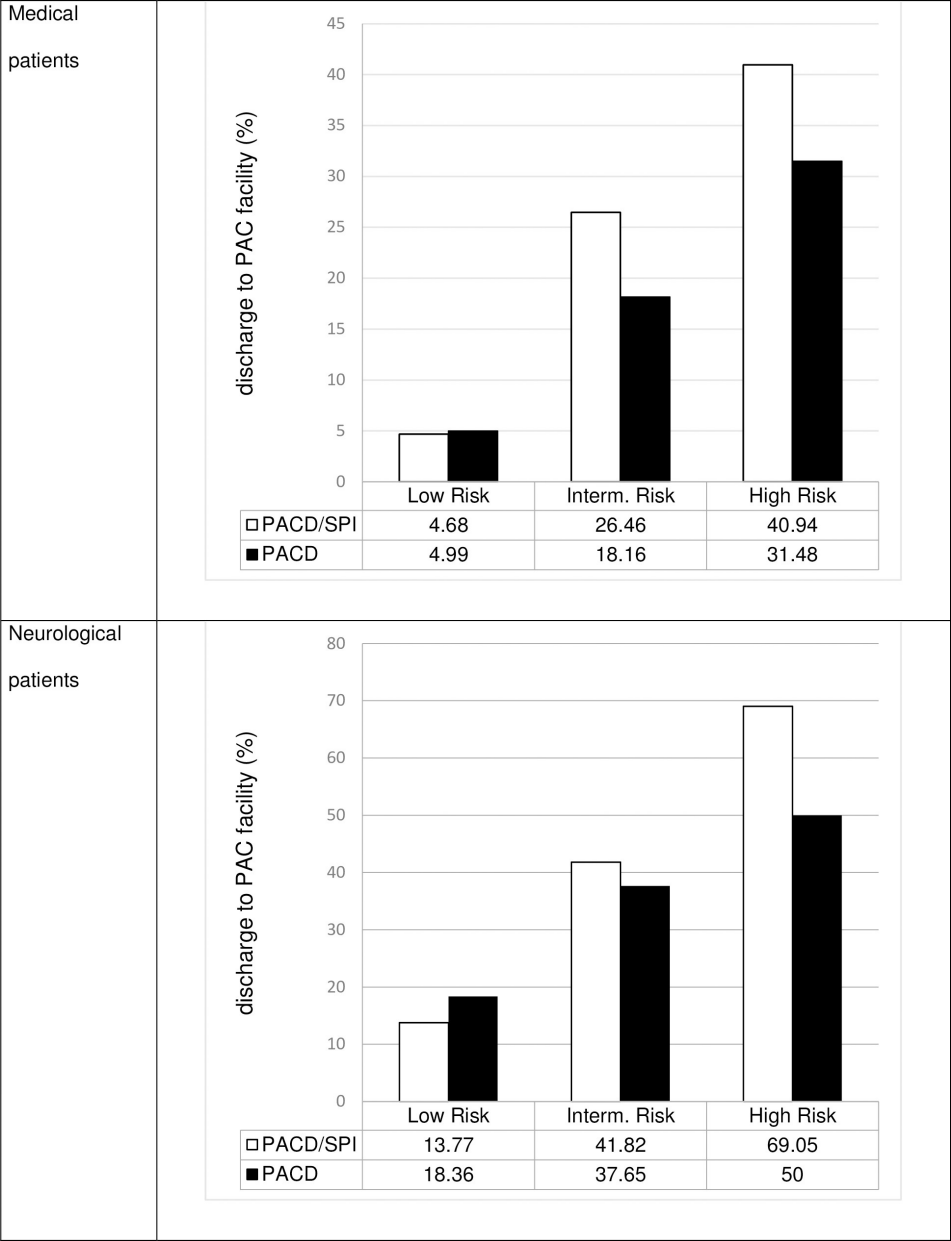
Percentage of patients discharged to PAC facility per risk group (PACD/SPI vs. PACD). Abbreviations: PAC: post-acute care; PACD: post-acute care discharge score; SPI: self-care index.

Fig 3 shows relative frequency for an event per risk group within the two models. The positive and negative predictive values of both scores and for all risk groups separate are shown in Table 5.

**Table 5:** Prognostic accuracy of the total score PACD resp. SPI compared to combined PACD/SPI.

**Medical patients**					
	Risk for PAC discharge	sensitivity	specifity	positive predictive value (%)	negative predictive value (%)
PACD	Intermediate+High Risk^a^	72.7%	65.4%	20.9%	95%
	High Risk	22.7%	93.8%	31.5%	90.6%
SPI	Risk^c^	64.0%	83.7%	32.8%	94.9%
PACD/SPI	Intermediate+High Risk^b^	68%	82%	32.3%	95.3%
	High Risk	34.7%	93.7%	40.9%	91.9%
**Neurological patients**					
	Risk for PAC discharge	sensitivity	specifity	positive predictive value (%)	negative predictive value (%)
PACD	Intermediate+High Risk^a^	40.4%	80.8%	39.2%	81.6%
	High Risk	6.4%	98.1%	50%	77.4%
SPI	Risk^c^	58.5%	85.1%	54.5%	87.0%
PACD/SPI	Intermediate+High Risk^b^	55.3%	85.4%	53.6%	86.2%
	High Risk	30.9%	95.8%	69%	81.9%

^a^ cut-off: intermediate ≥8; high: ≥16

^b^ cut-off: intermediate: >15; high: >25

^c^ cut-off: risk ≤32

Abbreviations: PAC: post-acute care; PACD: post-acute care discharge score; SPI: self-care index

Persons who left the hospital for an institution and persons who have returned home were evaluated separately. When SPI was added to the model, we found that in the group who experienced the event (n = 150), 34 subjects correctly moved up in a higher risk category, but 16 subjects incorrectly moved down to a lower risk category. NRI was 14.2% and significant (p<0.05), IDI was 4.83% (p<0.05). Of the subjects who did not experience the event (n = 1'192) 82 subjects were correctly classified in a lower risk category, whilst 56 subjects incorrectly moved up to a higher risk category (Table 6). Similar data was shown in the neurological group where 35 subjects in the group who experienced the event (n = 94) correctly moved up in a higher risk category, but 24 subjects incorrectly moved down to a lower risk category (Table 3B). NRI was 67.9% and significant (p<0.001), IDI was 18.85% (p<0.05). Of the subjects who did not experience the event (n = 308), 192 subjects were correctly classified in a lower risk category, whilst 19 subjects incorrectly moved up to a higher risk category (Table 7).

**Table 6:** Reclassification among medical patients who experienced a PAC facility discharge event and those who did not experience such a discharge event.

Model without SPI	Model with SPI			
Frequency (Row percent)	<15%	15–40%	> = 40%	Total
Events				
<15%	49	24	2	75
15–40%	10	39	8	57
> = 40%	0	6	12	18
Total	59	69	22	150
Non-Events				
<15%	954	30	2	986
15–40%	73	84	24	181
> = 40%	1	8	16	25
Total	1028	122	42	1,192

Abbreviation: SPI: self-care index

Highlighted green: cases correctly classified in a higher (events) or lower (non-events) risk category

Highlighted red: cases incorrectly classified in a higher (non-events) or lower (events) risk category

**Table 7:** Reclassification among neurological patients who experienced a PAC facility discharge event and those who did not experience such a discharge event.

Model without SPI	Model with SPI			
Frequency (Row percent)	<15%	15–40%	> = 40%	Total
Events				
<15%	3	3	1	7
15–40%	21	21	31	73
> = 40%	2	1	11	14
Total	26	25	43	94
Non-Events				
<15%	13	6	0	19
15–40%	182	74	13	269
> = 40%	4	6	10	20
Total	199	86	23	308

Abbreviation: SPI: self-care index

Highlighted green: cases correctly classified in a higher (events) or lower (non-events) risk category

Highlighted red: cases incorrectly classified in a higher (non-events) or lower (events) risk category

## Discussion

Patients’ self-care ability is a well investigated predictor for different adverse patient outcomes, such as a post-acute care deficit. Our results indicate that self-care abilities are also an appropriate predictor for the risk of PAC facility discharge. By adding the information of an assessment measuring the ability and impairments of patients’ self-care (SPI) on the ward, the AUC of the screening instrument PACD improved by 7.8% in the medical sample and 12.8% in the neurological. NRI showed that 22.6% (n = 34) and 37.2% (n = 35) of the patients in the event groups were correctly classified in a higher risk group, whilst 7.1% (n = 82) and62.3% (n = 192) in the non-event group were correctly classified in lower categories. This results in a higher positive predictive value for the combined score—overall risk groups (see Table 5). As with any screening instrument, some patients are not classified correctly. However, there is no disadvantage for falsely positively identified patients. The improved accuracy will help to identify patients at risk for a PAC facility discharge with a higher probability. Within the high risk group, almost one out of two patients will be discharged to a PAC facility. Since the SPI is widely used in German-speaking hospitals, no extra resources will be needed to complete the screening and benefit from results mentioned above.

Our study has limitations. Patients from only one institution (KSA) were included, restricted to medical and neurological patients, which limits generalizability to these groups. We have excluded patients transferred from or to another hospital as already done in the development study because these patients have not reached the primary endpoint of discharge to a PAC facility or home. However, because these patients are not in the focus of the screening instrument, we do not expect any influence on the validity or generalizability of the instrument. Because the data have been collected in real life, the PACD and/or SPI may be incomplete. We've found that patients with missing SPI/PACD data show systematic differences from patients with complete SPI/PACD data in terms of age (younger age) and length of stay (shorter length of stay). In addition, patients with missing SPI data are less likely to discharge to a PAC facility than patients with complete SPI data. No difference in gender could be observed between patients with complete and missing data. Based on these results, it cannot be clearly concluded whether the validity and transferability of the results have been affected. Because the PACD was validated exclusively for medical patients, data is missing for patients from other fields (e.g. surgery). Similar analysis could be performed to estimate the potential to identify patients in the need of post-acute care facility by the PACD with and without SPI in these fields. Based on the broad random sample, it is expected that the researched patients are representative for the medical patients in our clinic and therefore the transferability of the results is given. Nurses and physicians were not blinded to PACD and SPI scores and thus may have adapted their recommendation accordingly. This may overestimate the performance of the PACD/SPI. However, all data was collected as part of the clinical routine work, whereby no influence by the researchers was possible and therefore performance bias was prevented [20].

Regarding the presented results, showing a clearly distinct improvement for the current screening procedure, we intend to implement the combined score (PACD/SPI) in our clinical information system. Thereby, nursing staff will be informed within the medical record about patients with an intermediate or high-risk for PAC facility discharge—as soon as the PACD and SPI assessments are completed. High-risk patients will also be automatically reported to the in-house social workers for closer inspection. We expect a low single-digit amount of cases per week and therefore an acceptable extra effort. Future studies will have to evaluate whether this actually leads to more timely hospital departures.

## Conclusions

Incorporating an early assessment of patients’ actual intrahospital self-care ability (SPI) to the PACD led to an improved prognostic accuracy for identifying adult, medical and neurological patients at risk for discharge to a PAC facility.
